# Anti‐Prion Systems in *Saccharomyces cerevisiae*

**DOI:** 10.1111/jnc.70045

**Published:** 2025-03-25

**Authors:** Reed B. Wickner, Yuho Hayashi, Herman K. Edskes

**Affiliations:** ^1^ Laboratory of Biochemistry and Genetics National Institute of Diabetes and Digestive and Kidney Diseases, National Institutes of Health Bethesda Maryland USA

**Keywords:** Btn2, Cur1, Hsp104, inositol polyphosphates, ribosome‐associated chaperones, Upf proteins

## Abstract

[PSI+] is a prion (infectious protein) of Sup35p, a subunit of the translation termination factor, and [URE3] is a prion of Ure2p, a mediator of nitrogen catabolite repression. Here, we trace the history of these prions and describe the array of anti‐prion systems in *S. cerevisiae* . These systems work together to block prion infection, prion generation, prion propagation, prion segregation, and the lethal (and near‐lethal) effects of most variants of these prions. Each system lowers the appearance of prions 2‐ to 15‐fold, but together, ribosome‐associated chaperones, the Hsp104 disaggregase, and the Sup35p‐binding Upf proteins lower the frequency of [PSI+] appearance by ~5000‐fold. [PSI+] variants can be categorized by their sensitivity to the various anti‐prion systems, with the majority of prion isolates sensitive to all three of the above‐mentioned systems. Yeast prions have been used to screen for human anti‐prion proteins, and five of the Bag protein family members each have such activity. We suggest that manipulation of human anti‐prion systems may be useful in preventing or treating some of the many human amyloidoses currently found to be prions with the same amyloid architecture as the yeast prions.

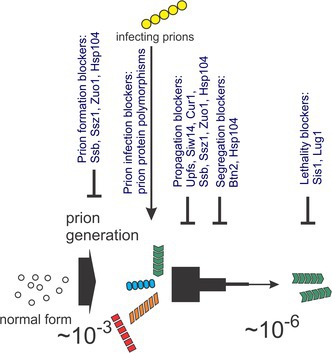

## Graphical abstract

Yeast pathogenic prions have the same in‐register parallel folded β‐sheet architecture as infectious amyloids of human transmissible spongiform encephalopathy, Alzheimer's disease, Parkinson's disease, and type 2 diabetes. Six anti‐prion systems in yeast (identified by their gene names) together lower the load of prions detected ~5000‐fold by blocking prion formation, segregation, and propagation. It is suggested that homologous or analogous human systems may provide new routes to deal with human amyloidoses.

**Abbreviations**
Aβ: peptide whose amyloid is central in Alzheimer's disease
BAG proteins: any of the 6 human proteins with a repeat “BAG” sequemce
Btn2p: a sequestrase curing most [URE3] prions
CJD: Creutzfeldt—Jakob disease (the TSE of humans—a PrP‐based prion disease)
Cur1p: protein of unknown function that cures [URE3]
DNAJA1: J‐protein (an Hsp40) most closely related to Ydj1p
DNAJB6: human J protein homologous to yeast Sis1p
Hsp104: a disaggregating chaperone necessary for prion propagation and recovery from heat shock; Hsp40 (e.g. Sis1p), Hsp70 (e.g. Ssa1, 2, 3, 4) and Hsp90 are other chaperone families
Hsp42: small chaperone curing [URE3] with Btn2
[LSB+]: metastable prion of Lsb2p
Lug1p: a substrate determining subunit of a ubiquitin ligase blocking [URE3] lethality
[PIN+]: prion of Rnq1p
prion: infectious protein without the need for a nucleic acid
PrP: a cell surface protein of mammals some of whose amyloid forms are infectious
Prp19: human homolog of yeast Prp19p, a U‐box E3 ubiquitin ligase
[PSI+]: prion of Sup35p
Siw14p: inositol polyphosphate‐5‐pyrophosphate pyrophosphatase that makes cells unable to propagate certain [PSI+] prions
Sse1p and Fes1p: nucleotide exchange factors for Hsp70s
Ssz1, Zuo1 and Ssb1,2: ribosome‐associated chaperones blocking propagation of several chaperones
Sup35p: subunit of the translation termination factor
TSE: transmissible spongiform encephalopathy (a prion disease of mammals)
Upf1,2,3: ribosome‐associated complex responsible for nonsense‐mediated mRNA decay
Ure2p: mediator of nitrogen catabolite repression in *S. cerevisiae* and the protein whose amyloid is the [URE3] prion
[URE3]: prion of Ure2p

## 1 Introduction

Yeast prions have contributed many unique aspects to the prion field. The early genetic evidence that [URE3] and [PSI+] are prions was of a type not possible in mammalian systems. The intimate involvement of chaperones in prion propagation was first documented in yeast systems. The in‐register parallel folded architecture first shown in yeast (but now realized for PrP‐based prions) brought a solution to the mystery of how proteins could template their own structure/conformation. The variety of cellular anti‐prion systems discovered in yeast suggests therapeutic approaches alternative to the elimination of prion protein monomers. The very existence of yeast prions as non‐chromosomal genes showed that proteins can be genes, that prions could mediate inheritance, and that the prion concept is not limited to transmissible spongiform encephalopathies, nor to amyloidoses.

## 2 Discovery of Yeast Prions

Infectious proteins were proposed as early as the 1960s to explain the transmissible spongiform encephalopathy (TSE) of sheep (scrapie) (Alper et al. 1966, 1967; Griffith 1967; Dickinson et al. 1968), but the proof of this notion would await many years, even beyond Prusiner's (1982) coining of the term “prion” based on his isolation of a protein (PrP) purifying with scrapie infectious material (Prusiner 1982) (which indeed was the protein causing TSEs). The history of scrapie, the sheep form of the TSEs, goes back to at least the mid‐18th century, with the disease named for the fact that the sheep scrape off much of their wool because they (apparently) feel “itchy” (M'Gowan 1914). It has been speculated that the Chinese character 痒, meaning “Itchy” (with疒, meaning ‘disease’, and 羊meaning ‘sheep’), derives from the existence of scrapie in ancient China (Wickner 2005). Whether the philology is right or not, prion diseases must be older than history, being based on very old gene sequences. The 129 V/M polymorphism of human PrP is believed to have been selected by the protective effect of heterozygotes against CJD, likewise indicating that TSEs are an ancient disease (Mead et al. 2003). Demonstration that PrP was encoded by the host (Chesebro et al. 1985; Oesch et al. 1985), the requirement of this protein for propagation of scrapie (Brandner et al. 1996), and the demonstration that mutations of the PrP gene could result in human TSE syndromes (Collinge et al. 1989; Hsiao et al. 1989) strongly supported the infectious protein model. The ability of protease‐resistant PrP from TSE‐infected animals to template in vitro conversion of protease‐sensitive PrP to a protease‐resistant form with the same specificity as infectivity (Kocisko et al. 1994, 1995) gave a concrete basis for the infectious protein model by demonstrating that templating of the protease‐resistance property was occurring. However, the controversy in the mammalian prion field was not over.

Meanwhile, in 1965, Brian Cox discovered a non‐Mendelian genetic element he named [PSI] that increased the efficiency of non‐sense suppression (Cox 1965), and in 1971 Francois Lacroute discovered [URE3], also a non‐chromosomal gene, in this case allowing *ura2* mutants to take up and grow on ureidosuccinate (the product of Ura2p = aspartate transcarbamylase) in place of uracil (Lacroute 1971).

Michel Aigle and Lacroute found that the [URE3] non‐mendelian gene required the *URE2* gene for its propagation and that the phenotype of the *ure2* mutants is the same as that of the presence of the [URE3] cytoplasmic element (Aigle and Lacroute 1975). This result seemed paradoxical (Wickner 1994) because chromosomal mutants unable to propagate the killer toxin–encoding M_1_ dsRNA (called *mak1*, etc. for maintenance of killer) always had the opposite phenotype (non‐killer (K−)) compared to strains with the M_1_ dsRNA (K+) (reviewed in Wickner et al. 2013). Similarly, chromosomal mutants unable to propagate mitochondrial DNA have the opposite phenotype of strains with the mitochondrial DNA. However, if [URE3] were an inactive form of Ure2p that was capable of catalyzing the conversion of normal Ure2p into this same inactive form, then both the *ure2* mutants and strains carrying [URE3] would have little or no normal Ure2p and have essentially the same phenotype (Wickner 1994). This “genetic criterion” should apply to all prions whose phenotype is due to deficiency of the normal protein but does not apply to a prion, like [PIN+], a prion of Rnq1p detected by its occasional cross–seeding to generate [PSI+], an activity not due to the absence of the normal form (Derkatch et al. 2001).

**FIGURE 1:**
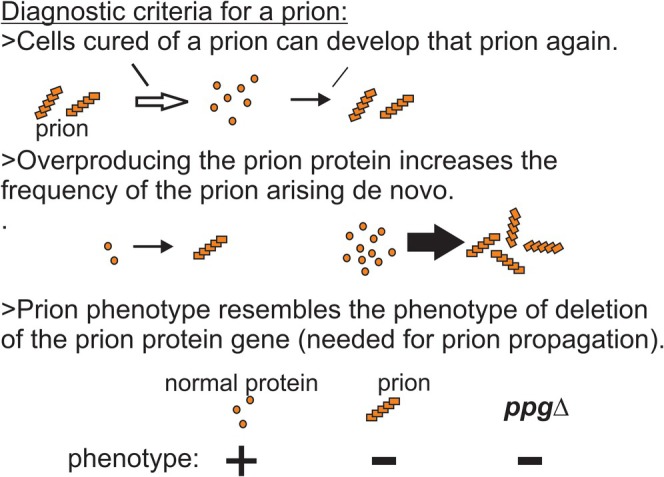
Genetic criteria for a prion. Three genetic properties that distinguish prions from nucleic acid‐based non‐chromosomal genetic elements (Wickner 1994). *ppg* = prion protein gene.

Two other properties were predicted for such a non‐chromosomal element that contrast with what was well‐known for viruses and plasmids (Figure 1) (Wickner 1994). First, transient overproduction of Ure2p should increase the frequency with which [URE3] arises simply because there would be more molecules with the potential to undergo the original change (whatever that change may be), and, by hypothesis, any changed molecules would catalyze the conversion of the normal molecules to the same abnormal form. Second, if one could cure (meaning efficiently eliminate, not just mutate) [URE3] by some treatment, it should still be capable of arising again de novo at some low frequency. Each of these properties was realized for [URE3]. Transient overproduction of Ure2p induced a 100‐fold increase in the frequency with which [URE3] arose, and curing [URE3] (by transient growth in the presence of 5 mM guanidine) produced cells that could still (at ~10^−5^ or 10^−6^) give rise to [URE3] (Wickner 1994).

These same three properties were already known for [PSI+] and Sup35 (Cox 1965; Lund and Cox 1981; Chernoff et al. 1993), and so it was proposed that [PSI+] was an infectious protein (a “prion”) of Sup35p (Wickner 1994). The clear parallel of the genetic properties of [URE3]/Ure2p and [PSI+]/Sup35p made a rather convincing case, although at that time we could not specify what was the nature of the self‐propagating change that was the basis of these genetic phenomena. The importance of the three genetic criteria for a prion lay in the fact that none of them were expected for a nucleic acid replicon, the alternative explanation for the non‐chromosomal genes [URE3] and [PSI+]. These criteria have been useful in the discovery of other yeast and fungal prions (Coustou et al. 1997; Patel et al. 2009; Suzuki et al. 2012; Chakrabortee et al. 2016). Recently, a prion of Cut4/Apc1 of *Schizosaccharomyces pombe* has been discovered (Sharma et al. 2024). Cut4 is a component of the anaphase‐promoting complex but also has a role in heterochromatin silencing at centromeres and the mating type locus (Dubey et al. 2009). A mutant of Cut4 results in a semidominant loss of silencing which has non‐Mendelian inheritance, reversible curing, induction by overexpression of Cut4, and other prion properties (Sharma et al. 2024).

That [URE3] was based on the amyloid of Ure2p was suggested by its partial protease resistance specifically in [URE3] cells (Masison and Wickner 1995). The aggregation of Sup35p in [PSI+] cells or extracts (Patino et al. 1996; Paushkin et al. 1996), the ability of the Sup35p prion domain to form amyloid in vitro (King et al. 1997), and the self‐propagation of the amyloid formation process (Glover et al. 1997; Paushkin et al. 1997) likewise suggested that amyloid formation was the basis of this prion. Amyloid formation by the Ure2 prion domain (Taylor et al. 1999) and in vivo aggregation of Ure2p specifically in [URE3] cells (Edskes et al. 1999) also supported an amyloid basis of this prion. The demonstration of infection with [PSI+] by amyloid of recombinant Sup35p (King and Diaz‐Avalos 2004; Tanaka et al. 2004) and infection with [URE3] by amyloid of Ure2p (Brachmann et al. 2005) ended any doubts. However, prions need not be amyloids. Specifically, vacuolar protease B is made as an inactive precursor protein, which is normally activated by cleavage by protease A (Jones 1991) but can be activated by active mature protease B (Zubenko et al. 1982). Thus, in mutants lacking protease A, active protease B is a non‐amyloid prion (called [BETA]) (Roberts and Wickner 2003). The [SMAUG+] prion based on Vts1p involves aggregation but not amyloid (Chakravarty et al. 2020).

Chernoff's finding that the disaggregating chaperone Hsp104 was essential for the propagation of [PSI+] (Chernoff and Ono 1992; Chernoff et al. 1995) started a large body of work detailing the central roles of Hsp70s, Hsp40s, Hsp90s (and co‐chaperones) and other protein chaperone and protein managing systems in the prion phenomena. This ongoing area has been ably reviewed (Liebman and Chernoff 2012; Winkler et al. 2012; Masison and Reidy 2015; Chernova, Wilkinson, et al. 2017; Matveenko et al. 2018; Berger et al. 2020; Masison et al. 2022) and some aspects will be discussed below.

## 3 The Central Mystery: How Do Prions Template Protein Structure?

One of the reasons for early skepticism that proteins could be infectious was that there was no means known by which proteins could template their own conformation. It was known that there were many “strains” of sheep scrapie, differing in their incubation period, the distribution pattern in the brain of the spongiform changes typical of the disease, and other aspects of the infection. Specifically, it was clear that the degree of protease resistance of PrP and the parts of the molecule that were resistant differed among biologically distinct prion strains (Bessen and Marsh 1992). Different conformational forms of the same protein were not a mystery, but how could the prion form template its conformation and induce the normal form to adopt the prion form?

In an effort to prove that there were sequences within the prion domains of Ure2p and Sup35p necessary for prion formation, the opposite conclusion was reached when it was found that all 5 random shuffles of either sequence left the protein able to form a prion (Ross et al. 2004; Ross, Edskes, et al. 2005). Thus, the sequences of the Sup35p and Ure2p prion domains are not important, but the amino acid content must be important. If the templating ability of the amyloids were due to a complementarity of amino acids in the interaction of a newly joining molecule with those in the filament, shuffling the sequence would surely eliminate the complementarity. However, an in‐register parallel folded beta sheet architecture features interactions of identical residues, and there is no reason why the order of the residues should destroy templating ability, as indeed it does not (Ross et al. 2004; Ross, Edskes, et al. 2005). This reasoning (Ross, Minton, et al. 2005) proved to accurately predict that these prion amyloid architectures are in‐register, folded, parallel β‐sheets (see below: Shewmaker et al. 2006; Baxa et al. 2007).

By varying the content of prion domains, it was found that charged residues were unfavorable, as expected, and hydrophobic residues favored prion formation (Toombs et al. 2010). Using these rules, changing just a few residues of marginally non‐prion proteins could make them capable of prion formation (Paul et al. 2015).

**FIGURE 2:**
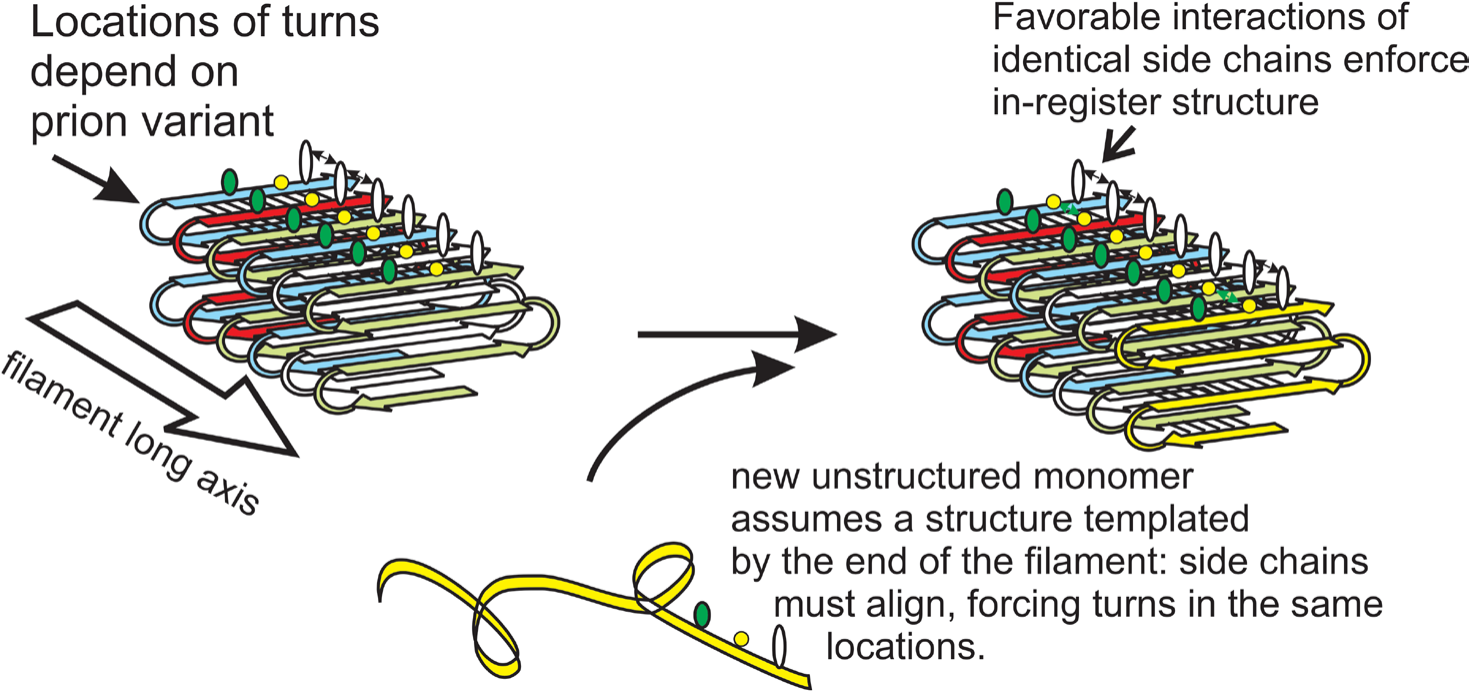
In‐register parallel folded β‐sheet architecture explains conformational templating. Using solid‐state NMR, it was shown that infectious amyloids of the prion domains of Ure2p, Sup35p, and Rnq1p have the folded parallel in‐register β‐sheet architecture diagrammed here. As discussed in the text, the positive interactions between identical (uncharged) amino acid side chains keep the structure in‐register. These same positive interactions drive the monomers joining the ends of the filaments to adopt the same conformation as the molecules already in the filament. This explains how a protein can template its conformation (Wickner et al. 2007, 2015). Figure from fig. 4 of Wickner et al. (2015).

Using solid‐state NMR, the infectious forms of the prion domains of two yeast prion proteins, Sup35p and Ure2p, were shown directly to have an in‐register parallel β‐sheet architecture (Shewmaker et al. 2006; Baxa et al. 2007), and this naturally suggested a templating mechanism (Wickner et al. 2007, 2015). The in‐register parallel architecture features a line of each residue parallel to the long axis of the filament (Figure 2), and it must be positive interactions among these identical side chains that keep the structure in‐register. For example, the presence of stabilizing hydrogen bonds between adjacent glutamine sidechains in this type of structure has been demonstrated (Chan et al. 2005). It was evident that the same interactions between identical side chains that stabilize the structure (hydrogen bonds between identical Q, N, S, or T residues; hydrophobic interactions between aligned identical L, V, I, F, Y, or W residues) should also direct a new molecule joining the end of a filament to adopt the same conformation as the molecules already in the filament in order to have these favorable interactions (Wickner et al. 2007, 2015). Of course, charged residues would have unfavorable (repulsion) interactions in such a structure, and there are very few charged residues in the Ure2p or Sup35p prion domains. Furthermore, mutations of residues in the prion domain from uncharged to charged often result in failure of prion propagation (Doel et al. 1994; DePace et al. 1998). This mechanism (Figure 2) seemingly solves the “propagation of strain information” mystery, but other mechanisms may be found to apply to other amyloid‐based prions. For example, the prion domain reported for the [MOD+] prion, Mod5p residues 194–215 (ITLKFDTLFLWLYSKPEPLFQR), has four (of 22) charged residues, two prolines, and only one Q or N residue (Suzuki et al. 2012). It would be of interest to know the structure of infectious amyloid of this peptide.

The parallel in‐register folded amyloid architecture was first recognized in peptide fragments of Aβ (Benzinger et al. 1998) and then in full‐length Aβ and several other human amyloid‐forming proteins (Balbach et al. 2002; Tycko 2006; Margittai and Langen 2008), including, recently, infectious PrP (Kraus et al. 2021; Manka et al. 2022).

## 4 [URE3] and [PSI+] Are Diseases of Yeast

While functional amyloids (e.g., Barnhart and Chapman 2006) and some beneficial prions (Saupe 2011; Chakravarty et al. 2020; Harvey et al. 2020) are known, [URE3] and [PSI+] are best understood as molecular diseases of yeast (reviewed in Wickner et al. 2015). Most variants of [URE3] or [PSI+] are very toxic or even lethal (McGlinchey et al. 2011), and even the mildest variants of either prion are so rare in the wild that they must convey a detriment greater than the 2%–3% growth slowing shown for the 2 μm DNA plasmid (Nakayashiki et al. 2005; Kelly et al. 2012). Nearly all yeast examined grow slower if they carry [URE3] (Wickner 1994). Reported benefits of [PSI+] (True and Lindquist 2000; Halfmann et al. 2012) have not been reproducible (Namy et al. 2008; Wickner et al. 2015). The Sup35p prion domain is important for the incorporation of the protein into phase‐separated particles in stressed cells, and [PSI+] makes cells slow in emerging from the stationary phase (Franzmann et al. 2018). The prion domain of Ure2p is important for the in vivo stability against degradation (Shewmaker et al. 2007).

## 5 Prion Variants

“Prion variants” is the expression used by yeast people for what researchers of mammalian prions call “prion strains” As discussed above, yeast prion variants are the same prion protein forming amyloids with different conformations, each of which can stably propagate. Variants are usually categorized based on the strength of the prion phenotype and the stability of prion propagation (Derkatch et al. 1996). There is a general correlation between these two prion characters, but unstable strong and stable weak [URE3] prion variants are also known (Brachmann et al. 2005). Prion variants also differ in their sensitivity to anti‐prion systems. In some cases, there is a correlation between prion seed number (as determined by the method of Cox et al. (2003)) and sensitivity to a particular anti‐prion system (e.g., Btn2 and [URE3] (Wickner et al. 2014)), but in other cases, there is no such relation (Gorkovskiy et al. 2017; Son and Wickner 2018).

## 6 Anti‐Prion Systems in Yeast

The pathogenic nature of the yeast prions [URE3] and [PSI+] led us to suspect that there must be anti‐prion systems developed by the cell. We and others have found that there are many such systems blocking generation and infection by prions, propagation and symmetric segregation of prions, and the pathologic effects of those prions that escape the other mechanisms. We sought such systems acting at normal protein levels, although several were first detected by their ability to cure prions on overproduction. The general pattern found was that prion appearance was much more frequent in the absence of one or more of these anti‐prion systems and that many of the prion isolates in a host mutant in an anti‐prion system were cured on restoration of the wild‐type gene.

## 7 Btn2 Is a Sequestering Protein Curing Most [URE3] Variants

**FIGURE 3:**
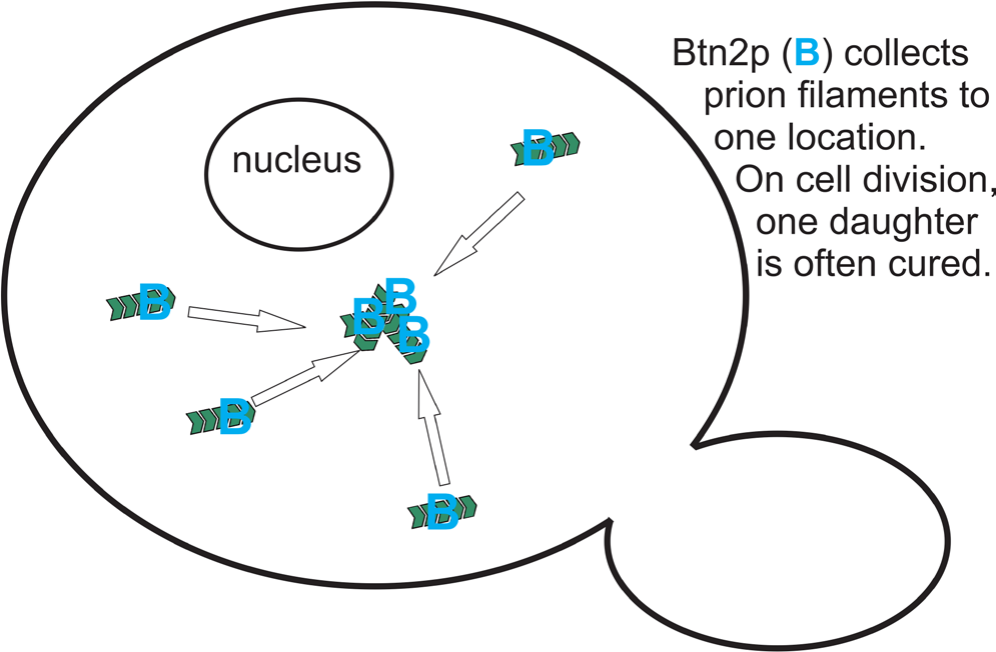
Btn2p collects prion amyloids (and other aggregates) limiting prion spread. In the course of curing [URE3], Btn2p‐RFP is found co‐localized with Ure2p‐GFP at a single place in each cell (Kryndushkin et al. 2008). This collection of Ure2p prion amyloids dramatically increases the likelihood of one of the progeny cells (mother or daughter) getting no amyloid and so being cured. Although paralogous to Btn2p, Cur1p does not co‐localize with Ure2p in the course of curing [URE3], and its mechanism of curing remains unknown.

Overexpression of either Btn2p or Cur1p was found to cure all variants of [URE3] (Kryndushkin et al. 2008). Either protein overexpressed in the absence of the other still cures [URE3] (Wickner et al. 2014). In [URE3] cells expressing Ure2p‐GFP and undergoing curing by Btn2p‐RFP, it was observed that the prion aggregates of Ure2p‐GFP that are dispersed in a [URE3] strain (Edskes et al. 1999) are instead collected at one place in the cell, co‐incident with the Btn2p‐RFP (Kryndushkin et al. 2008). The association of Btn2 with Ure2 aggregates does not require cell division, but the prion curing activity does (Kryndushkin et al. 2008). Neither Btn2 nor Cur1 changes the level of Ure2p in the cells, and blocking autophagy does not affect curing. These results support a sequestration model of prion curing; as cells divide, one daughter cell gets the sequestered prion amyloid and the other does not and is cured (Kryndushkin et al. 2008) (Figure 3). This sequestering activity of Btn2p has also been seen for non‐amyloid aggregates of VHL (von Hippel Lindau disease protein) (Malinovska et al. 2012) and non‐amyloid aggregates of optineurin (Kryndushkin et al. 2012). Optineurin is one of the proteins accumulating as aggregates in amyotrophic lateral sclerosis. Kryndushkin et al. found that optineurin aggregates precisely coincided with Btn2p. Overexpression of Btn2p partially relieved the toxicity of optineurin, while *btn2Δ* exacerbated its toxicity (Kryndushkin et al. 2012).

Hsp42 is a small chaperone and is necessary for the sequestration of some non‐amyloid aggregates but is reported not to be needed for amyloid aggregates of Rnq1p, the [PIN+] prion protein (Specht et al. 2011). However, Hsp42 overproduction cures [URE3] and helps Btn2p curing as well (Wickner et al. 2014).

To determine whether Btn2p and Cur1p acted in normal cells, [URE3] prions were formed in *btn2Δ cur1Δ* cells and then one or both genes were restored by mating with a wild‐type strain or by introducing plasmids. Over 90% of [URE3] prions formed in the double mutant were cured by the replacement of the normal genes. Replacement of either single gene destabilized those prions, again indicating that Btn2p and Cur1p act separately (Wickner et al. 2014). The [URE3] prion variants that were cured by normal levels of Btn2p and/or Cur1p were consistently those of low seed number, consistent with the sequestration mechanism of Btn2p curing (Wickner et al. 2014). Similarly, [PSI+] is not cured, even when Btn2p or Cur1p are overproduced perhaps because the seed number of the [PSI+] variants used was dramatically higher than that of [URE3] (Kryndushkin et al. 2008).

In addition to Btn2p's activity against prions in normal, unstressed cells, Btn2p and the small heat shock protein Hsp42p have both been shown to have roles in protein cleanup in DNA‐damaged, heat‐stressed, or ethanol‐exposed cells (Miller et al. 2015; Ho et al. 2019; Kato et al. 2019; Kumar et al. 2024). Btn2p sequesters denatured proteins in the stressed cells and also associates with the Hsp40 Sis1p and thereby with Hsp70s and Hsp104 to refold denatured proteins (Ho et al. 2019). The sequestering activity of Btn2 (Kryndushkin et al. 2008), the homology of Btn2 to HOOK1 (Kama et al. 2007) and the ability of HOOK proteins to move cargo via the microtubules (e.g., Walenta et al. 2001) suggests that Btn2 moves the prion amyloid by mediating its attachment to the cytoskeleton to collect and sequester the amyloid filaments to cure prions (Figure 3).

Cur1p curing does not act by sequestration, as tested by the same methods that gave clear co‐localization of Btn2p with Ure2p amyloid aggregates (Kryndushkin et al. 2008). Btn2p and Cur1p show clear sequence similarity, but not so high as to suggest identical mechanisms of function (Kryndushkin et al. 2008). Overexpression of Cur1p stabilizes weak variants of [PSI+] (unlike its curing of [URE3]) (Barbitoff et al. 2017). In one study, *btn2*Δ *cur1*Δ or *btn2*Δ did not destabilize [PSI+] but *cur1*Δ did mildly destabilize [PSI+] in heat‐pulsed cells (Barbitoff et al. 2017). Another study showed destabilization of [PSI+] in *cur1*Δ, *btn2*Δ, or *btn2*Δ *cur1*Δ unstressed cells (Son and Wickner 2022). If Btn2 cured [URE3] by relocating Sis1p to the nucleus, as suggested (Malinovska et al. 2012; Barbitoff et al. 2017), it would not explain Btn2's collecting filaments at one place in the cell (Kryndushkin et al. 2008; Kanneganti et al. 2011; Bezsonov et al. 2021). It is possible that the Cur1p curing is by a mechanism very different from Btn2, in spite of their sequence similarity.

### 7.1 Btn2 and Cur1 Degradation Suggests a Stress‐Induced Stress‐Remediating Activity

**FIGURE 4:**
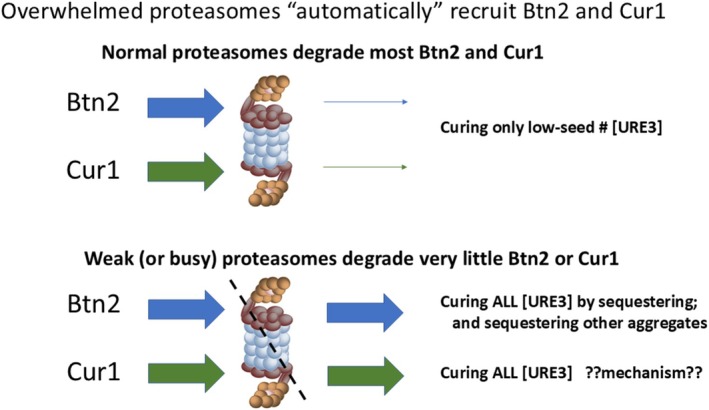
Proteasome regulation of Btn2p and Cur1p provides a “Plan B” for dealing with stress. Impairment of proteasome function by mutation produces massive increases in the levels of Btn2p and Cur1p (more than any of 4600 proteins examined) that cure [URE3] (Edskes et al. 2021). It is suggested that the accumulation of damaged proteins under stress conditions may overload the proteasomes, producing the same elevation of Btn2p and Cur1p which proteins aid in cleaning up the aggregates, a sort of “Plan B” for dealing with stress.

Among > 4600 yeast proteins examined, Btn2p and Cur1p are the most dramatically elevated in protein levels (> 200‐fold) by impairment of proteasome function (Edskes et al. 2021). A proteasome defect due to mutation of several assembly factor genes (*PBA1, ADD66, POC4, IRC25, HSM3*) or proteasome subunits (*RPN10, PRE9*) results in destabilization of [URE3], roughly in proportion to the given mutant's elevation of Btn2p and Cur1p (Edskes et al. 2021) (Figure 4). Btn2p is not specific for prions or amyloids but collects non‐amyloid aggregates of various proteins (Kryndushkin et al. 2012; Malinovska et al. 2012) as mentioned above. Clean‐up of proteins denatured by high ethanol conditions is delayed in *btn2*Δ cells (Kato et al. 2019). These results suggest that when proteasomes are overwhelmed with denatured proteins under some stress condition, their failure to degrade Btn2 and Cur1 will automatically provide more of these proteins to help clean up the mess (Ho et al. 2019; Edskes et al. 2021).

## 8 Upf Proteins Binding to Sup35p Produces an Anti‐[PSI+] Prion Effect

Screening the *S. cerevisiae* knockout collection revealed that Upf1p, Upf2p, and Upf3p have anti‐[PSI+] effects (Son and Wickner 2018). [PSI+] arises at 10–15 times the normal rate in *upf1*Δ, *upf2*Δ, or *upf3*Δ strains, and restoration of the defective gene cures the prion in over 90% of the isolates (Son and Wickner 2018). The Upf proteins are responsible for nonsense‐mediated mRNA decay, a process that recognizes a premature termination codon and destabilizes such mRNAs (reviewed by He and Jacobson 2015). Upf1, Upf2 and Upf3 form a complex on the ribosome that interacts with Sup35p (He et al. 1997, 2013; Czaplinski et al. 1998; Wang et al. 2001; Ivanov et al. 2008; He and Jacobson 2015). Mutational analysis of Upf1 and Upf2 showed that it was complex formation with Sup35p that affected prion propagation not nonsense‐mediated mRNA decay. Moreover, one‐tenth the molar concentration of purified Upf1p blocks amyloid formation of Sup35p in vitro (Son and Wickner 2018). It is suggested that the Upf complex competes with Sup35p amyloid filaments for available Sup35p monomers, thereby curing many [PSI+] variants. Alternatively, Upf proteins may block the ends of growing [PSI+] filaments with the same effect. Indeed, Upf1‐RFP decorates amyloid filaments of Sup35‐GFP, consistent with the latter hypothesis (Son and Wickner 2018). It should be generally the case that normal binding partners of a potential prion protein must compete with the pathologic interactions of the monomer prion protein with amyloid filaments. This provides a possible avenue of attack on any prion/amyloidosis whose normal binding partners have sufficiently tight interactions or can be made so.

## 9 Inositol Polyphosphates (IPs) Role in [PSI+] Prion Propagation

In the same screen that revealed a role for Upf proteins as anti‐[PSI+] components, Siw14p was found to have a similar effect (Wickner et al. 2017). Siw14p is a pyrophosphatase specific for inositol polyphosphates with a 5‐pyrophosphate (5PP‐IPs) (Steidle et al. 2016). In *siw14*Δ strains, cells have elevated levels of inositol 5‐pyrophosphates and are more resistant than wild‐type cells to several stress conditions, an effect mediated by the transcription factors Msn2 and Msn4 (Steidle et al. 2020). The *siw14*Δ strains are permissive for some variants of [PSI+] that cannot propagate in wild‐type cells, but the mechanism of this effect is unclear (Wickner et al. 2017). Mutants in the inositol polyphosphate biosynthetic pathway that block the environmental stress response (ESR) (*kcs1*Δ *vip1*Δ) and do not show the ESR (Worley et al. 2013) did not affect [PSI+] prion propagation (Wickner et al. 2017). It was also found that most [PSI+] variants do require some inositol pyrophosphates for their propagation (Wickner et al. 2017). This requirement can be met by the 5‐PP‐IPs or by IP_6_. Although the 5‐PP‐IPs support [PSI+] propagation, 1‐PP‐IPs appear to have an anti‐prion effect (Wickner et al. 2017).

## 10 Ribosome‐Associated Chaperones Have Anti‐Prion Activity Against Several Prions

Ssb1 and the nearly identical Ssb2 (Hsp70 family), Ssz1 (Hsp70), and Zuo1 (Hsp40) are ribosome‐bound chaperones whose function is to ensure proper folding of nascent proteins as they emerge from the ribosome (Nelson et al. 1992; Pfund et al. 1998; Yan et al. 1998; Gautschi et al. 2001, 2002) (reviewed in Zhang et al. 2017; Deuerling et al. 2019). Mutants lacking Ssb1/2 or Ssz1, or Zuo1 have similar phenotypes, including slow growth, hypersensitivity to translation inhibitors, and accumulation of protein aggregates.

Chernoff showed that *ssb1*Δ *ssb2*Δ formed the [PSI+] prion at > 10‐fold the wild‐type frequency (Chernoff et al. 1999), and similar effects have been seen for *zuo1*Δ and *ssz1*Δ strains (Amor et al. 2015; Kiktev et al. 2015; Son and Wickner 2020). In our experiments, restoring the defective chaperone cured a majority of the prions isolated in any of the mutants, indicating that the ribosome‐associated chaperones interfere with the propagation of most [PSI+] variants (Son and Wickner 2020). These chaperones also shape the array of prion variants that are found for [PSI+] (Son and Wickner 2020) and for [URE3] (see below; Jay‐Garcia et al. 2023).

The effects of the ribosome‐associated chaperones on prions were first found for [PSI+] (Chernoff et al. 1999) but are now known to be more general (Jay‐Garcia et al. 2023). [URE3] formation is increased five‐fold in *zuo1*Δ cells, and the stability of [URE3] isolates (though not the frequency of generation) is increased in *ssb1/2*Δ strains (Jay‐Garcia et al. 2023). In both cases, restoring the missing chaperone destabilized most of the prions isolated in the mutant but not [URE3] prion variants isolated in wild‐type cells. [LSB+] is a metastable prion of Lsb2, formed on artificial overproduction of Lsb2 or on heat shock, which also induces expression of Lsb2 (Chernova, Kiktev, et al. 2017). In *ssb1/2*Δ cells, Lsb2 levels are elevated and both the frequency and stability of [LSB+] are increased, and the induction of [LSB+] prions by heat shock is massively increased (Jay‐Garcia et al. 2023).

## 11 Hsp104, a Disaggregating Chaperone, has Anti‐Prion Action

The hexameric Hsp100‐family member, Hsp104, has two actions on yeast prions (Chernoff et al. 1995). Deletion of Hsp104 results in the loss of all of the amyloid‐based yeast prions, but transient overexpression of Hsp104 efficiently cures [PSI+] and weakly cures [URE3]. We refer to these two aspects as the prion propagation function and the prion curing function of Hsp104, respectively. The propagation function of Hsp104 is its ability, in cooperation with Hsp70s and Hsp40s (e.g., Higurashi et al. 2008; Reidy et al. 2012), to extract prion protein monomers from a filament, threading the molecule through the Hsp104 hexamer (Lum et al. 2004; Weibezahn et al. 2004), thereby breaking the filament in two and producing a new prion seed. Unlike the propagation activity, the overproduction curing activity requires the activity of Hsp90 and the Hsp90 co‐chaperone Sti1p (Moosavi et al. 2010; Reidy and Masison 2010). The mechanism of the overproduction curing remains controversial, with suggestions including (1) overactivity of the monomer extraction activity, (2) asymmetric segregation of prion filaments, and (3) non‐productive binding to filaments, preventing the binding of other chaperones needed for productive (filament‐cleaving) binding (Helsen and Glover 2012; Ness et al. 2017; Stanford et al. 2023).

Hsp104 mutants retaining the filament‐cleavage activity (as judged by stable prion propagation and heat shock resistance) but lacking the prion‐curing activity have enabled studies of the roles of the two Hsp104 functions (Hung and Masison 2006). Many [PSI+] variants generated in one such mutant, *hsp104*,^*T160M*^ were lost when the wild‐type gene was restored, showing that Hsp104 has an anti‐prion activity (Gorkovskiy et al. 2017). Like the overproduction curing activity, the anti‐prion activity of Hsp104 requires Sti1p and Hsp90 (Gorkovskiy et al. 2017).

## 12 Hsp90 and Its Cochaperones

Hsp90 is encoded by two genes in *S. cerevisiae, Hsp82* and *Hsc82*, at least one of which is essential for growth. Hsp90 works with co‐chaperones Aha1, Hch1, Cpr6, Cpr7, Sba1, and Sti1 to carry out a cycle of substrate binding release and ATP binding, hydrolysis, and release that maintains or restores the proper folding of certain “client” proteins. In addition to its role in Hsp104 overproduction curing (above), Lancaster et al. have shown that deletion mutants *hsc82*Δ, *aha1*Δ, *cpr6*Δ, *and cpr7Δ* convert a “weak” variant of [PIN+] to a strong variant, while *sba1*Δ converts a strong variant to a weak variant (Lancaster et al. 2013). Transfer of the altered [PIN+] to a wild type strain not carrying any prion ([pin‐]) produced cells with the altered [PIN+] phenotype, showing that the [PIN+] variant itself had changed (Lancaster et al. 2013). This phenomenon is related to the anti‐prion activities described above but differs in significant ways. The strong and weak [PIN+] variants were isolated in a wild type strain and transferred to each mutant. The mutant selected out a variant that was part of the “cloud” of variants present even in a theoretically pure strain (Bateman and Wickner 2013). In such cases, although the variant has changed, it usually remains capable of propagating in its host of origin. It would be of interest to examine whether [PIN+] generated in one of the mutants was lost on transfer to the wild type or on transfer to a different mutant.

## 13 Anti‐Prion Systems Cooperate to Block >99.9% of [PSI+] Prions

Single mutants in the anti‐prion systems show elevations of 2x to 15x in the frequency of appearance of the affected prions (Table 1). Blocking combinations of anti‐prion systems showed that the single mutants were not blocking a single anti‐prion system with variable efficiency but rather represent an array of different systems, acting independently (Son and Wickner 2022). Combining *ssz1*Δ, *hsp104*Δ and *upf1*Δ results in up to a~5000‐fold increase in the frequency with which [PSI+] arises (Son and Wickner 2022). Restoration of any one of these three genes (without restoring the other two) destabilized most of the [PSI+] prions arising in the triple mutant. Thus, these three systems act independently: each can cure in the absence of the other two. Moreover, the large majority of the prion variants arising in the triple mutant are never seen among prions arising in a wild‐type strain or even in the single mutants. Detailed analysis showed that while most of the increase in [PSI+] appearance is explained by the failure of the triply defective cells to cure prions sensitive to one or more of the anti‐prion systems, these systems also repress by up to 500‐fold the generation of prions that are not cured by normal levels of the anti‐prion proteins (Son and Wickner 2022). These experiments also bring into view the large number of prion variants and thus amyloid folds that Sup35p can form.

**TABLE 1:** Anti‐prion systems.

Anti‐prion gene(s)	Prion(s) affected	Activity	Action on prions: Blocks ____
*SSB1/2, SSZ1, ZUO1*	[PSI+], [URE3], [LSB]	Refolding nascent proteins	Generation and propagation
*HSP104*	[PSI+]	Disaggregating chaperone	Generation and propagation
*BTN2*	[URE3]	Sequestration of amyloids or aggregates	Segregation by sequestering amyloid
*CUR1*	[URE3]	Unknown	Propagation
*SIW14*	[PSI+]	Pyrophosphatase active on 5‐pyrophosphate‐inositolpolyphosphate	Propagation
*UPF1,2,3*	[PSI+]	Binding Sup35p (nonsense‐mediated mRNA decay)	Generation and propagation
*SIS1*	[PSI+]	Hsp40 J‐protein; protein renaturation with Hsp104, 70; filament cleavage	Lethality by solubilizing Sup35p from amyloid
*LUG1*	[URE3]	E3 ubiquitin ligase	Lethality

*Note:* We define “anti‐prion systems” as those cellular components that cure prions or block their adverse effects at normal levels of the anti‐prion protein(s). See text for more details and references.

**FIGURE 5:**
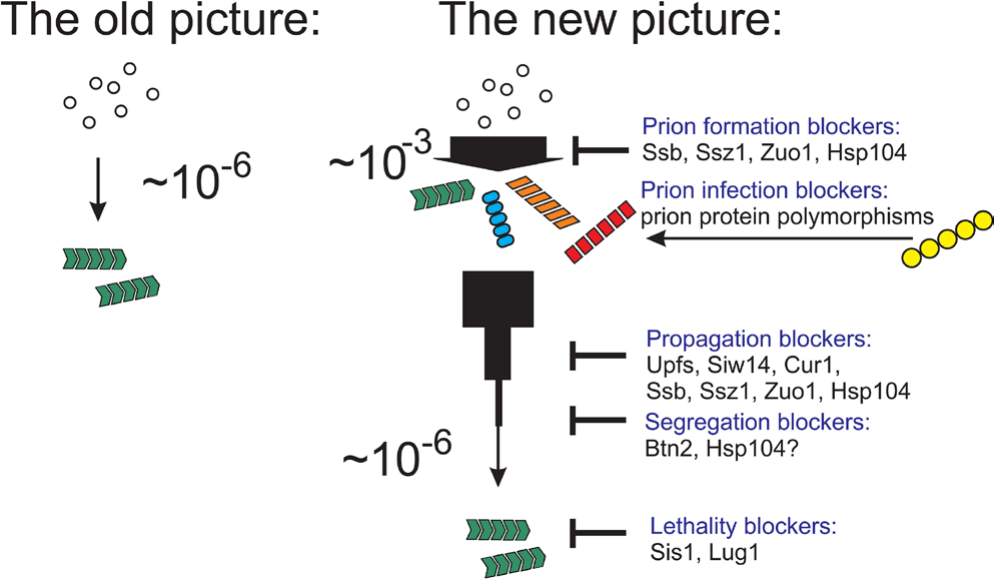
The changed landscape of prion biology. In the Old Picture, prions arose very rarely, and once generated, the cell/organism had little defense against their propagation or detrimental effects. In the New Picture, prions arise thousands of times more frequently, but nearly all prions are eliminated by an array of different anti‐prion systems acting on generation, propagation, and segregation. Limitations on infection, in the form of inter‐species and intra‐species barriers due to sequence differences between donor and recipient, have been known since before prions were “prions”. In addition, cellular components limiting the pathology produced by prions are now known. Gene names are listed in the abbreviations footnote. Figure modified from fig. 4 of Wickner et al. (2021).

The unexpected superabundance of [PSI+] prions arising in multiply defective strains and the results of the analysis of these novel variants have dramatically changed our view of the overall prion generation/propagation/infection process (Figure 5). In the old picture, prions arise or infect extremely rarely and if a species barrier (or intraspecies barrier) is overcome, the host has little resistance to the effects of the prion, whether it be the devastating CJD, the slower (but still ultimately devastating) effects of Alzheimer's disease, or the (varying from lethal to mild) effects of yeast prions. In contrast, the new picture features thousands of times higher prion generation frequency but an array of anti‐prion systems producing a highly effective (though not perfect) culling of nearly all prions arising.

## 14 Sis1p and Lug1p Block the Pathology of [PSI+] and [URE3], Respectively

Sis1p is the Hsp40 that acts, with Hsp70s and Hsp104, to cleave amyloid filaments in prion propagation (Higurashi et al. 2008). In studying the role of Sis1p domains in prion propagation and cell growth, it was found that Sis1JGF, including only the J domain involved in interaction with Hsp70 and the GF (glycine‐phenylalanine rich) domain, is sufficient to support cell growth and to propagate the [PSI+] prion, but in such cells, strong variants of [PSI+] are lethal (Kirkland et al. 2011). The lethality of [PSI+] is due to near complete sequestration in amyloid filaments of the essential Sup35p and is suppressed by expressing Sup35C (Kumar et al. 2021), which lacks the prion domain and so is not incorporated into the amyloid filaments. The Sis1JGF, lacking several C‐terminal domains, makes the Hsp104‐Hsp70‐Sis1 complex inefficient in removing monomers from the amyloid filaments and allowing their refolding.

A transposon mutagenesis screen for genes preventing [URE3] pathogenesis showed that *LUG1* (*YLR352W*) is essential only in the presence of the [URE3] prion (Edskes et al. 2018). Lug1 is an F‐box protein (Seol et al. 2001), meaning it is one of ~20 substrate‐determining subunits of an SCF ubiquitin ligase, a complex consisting of Skp1 (to which the F‐box binds), a cullin (a framework subunit), the RING protein Hrt1, Cdc34 (the catalytic subunit), and one of the F‐box proteins (Jonkers and Rep 2009; Hua and Vierstra 2011). However, the target(s) specified by Lug1 for ubiquitination have not yet been defined. The growth defect of *lug1*Δ [URE3] strains is absolute on non‐fermentable carbon sources and is seen with *lug1*Δ *ure2*Δ strains as well. Thus, it is the absence of normal Ure2p rather than the presence of the prion amyloid that is causing the failure of growth, but *lug1*Δ strains do not grow slowly on proline as the nitrogen source, a condition known to turn off Ure2p's nitrogen catabolite repression function. The growth defect is suppressed by either overexpression of Hap4 (a positive transcription factor for many mitochondrial proteins) or by *gln1* (glutamine synthase) mutations. Perhaps, Lug1 prevents an insufficiency of mitochondrial function when an excess of glutamine occurs. However, further work will be required to clarify the mechanism of these effects.

In the same screen (Edskes et al. 2018), transposon insertions in many chaperone‐encoding genes were found to be rare in [URE3]‐containing cells. In some cases, the encoded chaperone is known to be necessary for the propagation of [URE3]: Hsp104 (Moriyama et al. 2000), Ssa2p (Roberts et al. 2004; Sharma and Masison 2008), Sse1p (Kryndushkin and Wickner 2007), Fes1p (Kryndushkin and Wickner 2007), and Cpr7p (Kumar et al. 2015) are in this category. However, Hsp90s (Hsp82, Hsc82) and their cochaperones (Sti1, Sba1), J‐proteins (Ydj1, Caj1), “small chaperones” (Hsp42, Hsp26), and ribosome‐associated Hsp70s (Ssb1, Ssb2) also were rarely mutated in the [URE3] cultures compared to those without the prion. All of these components are known to not be necessary for the propagation of [URE3], and Ydj1 overexpression actually cures [URE3]. [URE3] is more toxic to cells than is the absence of Ure2p in a *ure2*Δ strain (McGlinchey et al. 2011; Edskes et al. 2018), and these chaperones must have roles in minimizing that stress. This is precisely the anti‐pathogen effect that the transposon screen was designed to detect.

## 15 Prion Protein Polymorphisms Block Prion Infection

Mouse‐adapted scrapie cannot infect hamsters. That sequence differences of PrP were the basis of the “species barrier” was established by transgenic mouse experiments (Prusiner et al. 1990). Similar results for interspecies transmission have been shown for yeast prions (Chernoff et al. 2000; Kushnirov et al. 2000; Santoso et al. 2000; Edskes and Wickner 2002; Baudin‐Baillieu et al. 2003; Chen et al. 2007). Interspecies mating among different *Saccharomyces* species (*paradoxus, mirakii*, etc.) is unusual, and the meiotic products of such matings are nearly all inviable. It is intra‐species mating that is the main driver of prion spread. Sequence differences among the Sup35p of various strains of *S. cerevisiae*, largely limited to the N and M domains (Jensen et al. 2001), produce an “intraspecies barrier” to [PSI+] transmission (Bateman and Wickner 2012). This intraspecies barrier is prion variant specific, and mutation and segregation of such variants have been documented (Bateman and Wickner 2013).

[URE3] also is limited in its transmission to strains with differing Ure2p sequences (Edskes and Wickner 2002; Edskes et al. 2009). As tested in *S. cerevisiae* , [URE3] generated with *S. paradoxus, S. bayanus, S. mikatae*, or *S. cariocanus* Ure2p each shows better transmission to cells with the same Ure2p than they do to cells expressing a Ure2p from another species (Edskes et al. 2009). The interspecies barrier for [URE3] is prion variant–specific as well. The Ure2p of *S. castelliae* does not form a prion (Edskes et al. 2009).

Similarly, the 129 M/V polymorphism of human PrP largely protects heterozygous individuals from all forms of CJD and is suggested to have been selected on that basis in an era when cannibalism was more common (Mead et al. 2003).

## 16 Human Proteins Curing Yeast Prions

Yeast can be useful in several ways in finding possible avenues for treatment of human amyloidoses/prions. In one approach, drugs were screened for their ability to cure yeast prions, and among those active on both [PSI+] and [URE3] were drugs that cure mammalian prions in tissue culture (Bach et al. 2003). Two such drugs proved to be active against a chaperone activity of ribosomal RNA (Blondel et al. 2016). DNAJB6 is the closest human homolog to Sis1p and was shown to block aggregation of polyQ proteins in animal cells (Hageman et al. 2010). DNAJB6 also blocks propagation of several yeast prions (Reidy et al. 2016) and can relieve the toxicity of some prions that it cannot cure (Dolder et al. 2022). The toxicity relief requires a serine/threonine‐rich amyloid‐binding region (Mansson et al. 2014). This was part of the motivation for a third approach, namely screening for human proteins whose transient expression in yeast cures yeast prions (see below; Wu et al. 2023). In a fourth approach, knowing that the yeast ribosome‐associated chaperones block both the generation and propagation of many prions (see above), Kelly et al. have shown that the mammalian homologs of Zuo1 and Ssz1 (Mpp11 and Hsp70L1) can inhibit the generation of the [PSI+] prion (Kelly et al. 2023).

We made a bank of human ORFs in a yeast vector with a *GAL* promoter to detect human proteins whose transient expression in yeast can cure yeast prions (Wu et al. 2023). We found 19 proteins whose expression cured either [URE3] or [PSI+] or both (Table 2). We focused on those human proteins with close yeast homologs, assuming that such cases were more likely to be acting in yeast as they would in human cells.

**TABLE 2:** Human proteins curing yeast prions.

Human gene name	Overexpression curing		Yeast homolog gene				
			Yeast homolog	Overexpression curing		Deletion curing	
	[PSI+]	[URE3]		[PSI+]	[URE3]	[PSI+]	[URE3]
BAG5	+	+	*SNL1*	−	+ (BD)^a^	−	−
BAG4	−	+					
PATL1	+	+	*PAT1*	−	−	−	−
DNAJA1	+	−	*YDJ1*	Enhanced^b^	+^c^	−	−
PRPF19	−	+	*PRP19*	−	−	Inviable	
KRI1	−	+	*KRI1*	−	−	Inviable	

*Note:* Human proteins curing the [PSI+] and [URE3] prions and that have yeast homologs are shown, with the effects of the human and yeast proteins shown. In addition, the following proteins were found in our screen to cure [URE3] (Wu et al. 2023): ZBTB20, AHI1, PCH3, CGN, PARS2, CCDC11, MAP11, CLCN3, BOLL, DAZ3, RBMY1F, OR51M, ALS2CL, PDE10A. BAG5, Bcl‐2‐associated athanogene 5, is a co‐chaperone of Hsp70s and is involved in autophagy. PATL1, Processing Body mRNA Decay Factor, is a component of P‐bodies. DNAJA1, DnaJ heat shock protein family (Hsp40) member A1. PRPF19, Pre‐mRNA‐processing factor 19, E3 ubiquitin ligase. KRI1, Essential nucleolar protein required for 40S ribosome biogenesis. Human proteins were expressed from the *GAL1* promoter in pH 1375. Yeast homologs were overexpressed from the *GAL1* promoter.

^a^ Data from Kumar et al. (2014).

^b^ Data from Barbitoff et al. (2017).

^c^ DATA from Moriyama et al. (2000); from Wu et al. (Wu et al. 2023).

DNAJA1 is a J‐protein (an Hsp40) most closely related to Ydj1p. While DNAJA1 expression cures [PSI+] and not [URE3] (Wu et al. 2023), overexpression of Ydj1p cures [URE3] (Moriyama et al. 2000) and not the strong [PSI+] used by Wu et al. (Sharma et al. 2009). Ydj1p curing is through its N‐terminal J‐domain, competing with Sis1p for interaction with Hsp70s in the Hsp104 –Hsp70 –Hsp40/J‐protein filament cleaving reaction (Sharma et al. 2009; Reidy et al. 2014). Mutation of the conserved HPD (to HPN) motif of Ydj1p eliminates its ability to bind to Hsp70 and to cure [URE3] (Sharma et al. 2009). The same D → N mutation in the HPD of the DNAJA1 J‐domain eliminates its [PSI+]‐curing activity (Wu et al. 2023), indicating that the human protein cures by a mechanism similar to its yeast homolog.

Human Prp19 is a homolog of yeast Prp19, the latter being an E3 ubiquitin ligase and an essential component of the U2 RNA splicing complex. Human Prp19 has a U‐box, a domain characteristic of a group of E3 ubiquitin ligases. Human Prp19 cures [URE3], and the deletion of its U‐box eliminates the prion curing activity, suggesting that the curing occurs via ubiquitination (Wu et al. 2023).

Two BAG proteins were detected in our screen; 5 of the 6 human BAG‐domain proteins could cure [URE3] and one cured [PSI+] (Wu et al. 2023). BAG proteins are defined by a 110 to 130 residue sequence with one copy in each of Bag1, 2, 3, 4, and 6 and five copies in Bag5 (reviewed in Takayama et al. 1999). We found that proteins with a single BAG domain could cure [URE3], while multiple BAG domains were needed to cure [PSI+]. Although most BAG proteins affect autophagy in mammalian cells (reviewed in (Pattingre and Turtoi 2022), blocking yeast autophagy did not affect the prion curing of BAG proteins, although the seed number of the yeast prions was diminished (Wu et al. 2023). BAG proteins bind to the nucleotide binding domain of mammalian Hsp70s and stimulate or inhibit ATPase activity (Hohfeld and Jentsch 1997; Takayama et al. 1997). We found that BAG proteins also bound to yeast Hsp70s and blocked the filament cleavage reaction as judged by an increased size of Sup35p amyloid filaments and the decrease in the “seed number” (= propagon number/cell) (Wu et al. 2023). The curing of yeast prions by BAG proteins is suppressed by overproduction of Sis1p (Wu et al. 2023). Sis1p and BAG proteins are both known to bind to the nucleotide binding domain of Hsp70s. These results suggest that BAG proteins cure by blocking Sis1p from binding to Hsp70s, thereby inhibiting the filament cleavage by the Hsp104–70–40 combination that is essential for prion propagation.

## 17 Prion Features of Common Human Amyloid Diseases

The major human amyloidoses, Alzheimer's disease, Parkinson's disease, and type II diabetes, each feature accumulation of amyloid that has the same in‐register parallel folded β‐sheet architecture (Antzutkin et al. 2000; Der‐Sarkissian et al. 2003; Jayasinghe and Langen 2004; Tycko 2006; Luca et al. 2007; Tuttle et al. 2016) as the three yeast prions that have been studied (see references above) and fully infectious PrP of mouse scrapie prions (Kraus et al. 2021). Over the last 10 years, strong evidence for actual infectivity of amyloid of each of these diseases has accumulated (Jaunmuktane et al. 2015; Prusiner et al. 2015; Mukherjee et al. 2017; Kim et al. 2019; Sampson et al. 2020). Iatrogenic infectivity of an Alzheimer's disease variant characterized by perivascular deposits of Abeta amyloid has been recognized in patients who had received cadaver‐derived human growth hormone (Banerjee et al. 2024), and statistical evidence for transmission of Alzheimer's disease via transfusion has been reported (Zhao et al. 2023).

## 18 Perspective

The study of yeast prions quickly broadened the prion field and deepened its understanding both with regard to the nature of the prion itself and the effects of other cellular components on prion processes. In several cases, human proteins curing yeast prions use mechanisms similar to their action in human cells, suggesting that screening in yeast may be useful in detecting human anti‐prion systems. The close human homologs of known yeast anti–prion proteins often have anti‐prion activity. It is hoped that these approaches will enable utilization of human anti‐prion systems to manage or prevent human amyloidoses in the same way that we manipulate the innate, humoral, and cellular immune systems to deal with viral, bacterial, and parasite infections.

## Author Contributions

**Reed B. Wickner:** conceptualization, methodology, writing – review and editing, writing – original draft, supervision, investigation. **Yuho Hayashi:** writing – review and editing, conceptualization, investigation. **Herman K. Edskes:** conceptualization, methodology, writing – review and editing, investigation.

## Conflicts of Interest

The authors declare no conflicts of interest.

## Data Availability

Data sharing is not applicable to this article as no new data were created in this study.
